# Sars-Cov-2 Infection in Patients on Long-Term Treatment with Macrolides in Spain: A National Cross-Sectional Study

**DOI:** 10.3390/antibiotics10091039

**Published:** 2021-08-25

**Authors:** Carmen Marina Meseguer Barros, Natalia Alzueta Isturiz, Rita Sainz de Rozas Aparicio, Rafael Aguilella Vizcaíno, Laura López Esteban, Sonia Anaya Ordóñez, Itxasne Lekue Alkorta, Salvadora Martín Suances, Jorge Ignacio Jiménez Arce, Maite Fernández Vicente, Yolanda Borrego Izquierdo, Raquel Prieto Sánchez, Silvia Casado Casuso, Rosa Madridejos, Carmen Marquina Verde, Rosa Tomás Sanz, María Oro Fernández, Sara Gallardo Borge, Eva Lázaro López, María Belén Pina Gadea, Mercedes Pereira Pía, María Victoria Maestre-Sánchez, Esther Ribes-Murillo, Constanza Gómez de Oña, Álvarez María Jesús Lallana, Concepción Celaya Lecea, María Ana Prado Prieto, Aranzazu Aranguez Ruiz, Vicente Olmo Quintana, Noemí Villén Romero, Carolina Payá Giner, Angeles Lloret Callejo, Alvaro Fernández Ferreiro, Blanca Basagoiti Carreño, Ana Aurelia Iglesias Iglesias, Antonio Martín Alonso, Ana Díez Alcántara, Esther Marco Tejón, Marta Lestón Vázquez, Mª Ángeles Ariza Copado, Marta Aparicio Cueva, Belén Escudero Vilaplana, Marisa Nicieza, Gracia Picazo Sanchiz, Genma María Silva Riádigos, Lucía Jamart Sánchez, Ángel García Álvarez, Antonio García Bonilla, Rafael Herrero Delicado, Virginia Arroyo Pineda, Belén de la Hija, Amelia Troncoso Mariño, Isabel Tofiño González, Mónica Susana Mateu García, Pablo García Vázquez, Joaquín Pérez Martín, Rocío Fernández-Urrusuno, on behalf of the Infectious Diseases SEFAP Team

**Affiliations:** 1 Service of Pharmacy, Ouest Primary Health Care Area, Madrid Health Service, Madrid C/Alonso Canonum 8, 28933 Madrid, Spain; carmen.meseguer@salud.madrid.org (C.M.M.B.); belenmaria.escudero@salud.madrid.org (B.E.V.); gracia.picazo@salud.madrid.org (G.P.S.); genmamaria.silva@salud.madrid.org (G.M.S.R.); lucia.jamart@salud.madrid.org (L.J.S.); 2 Unit of Drug Assessment, Advice and Research, Navarra Health Service, Pamplona, Plaza de la Paz s/n, 31002 Pamplona, Spain; natalia.alzueta.isturiz@navarra.es; 3 Service of Pharmacy, Cruces University Hospital, Osakidetza, Barakaldo, Vizcaya, Plaza Cruces s/n, 48903 Barakaldo, Spain; RITAMARIA.SAINZDEROZASAPARICIO@osakidetza.eus; 4 Service of Pharmacy, North Primary Health Care Area, Madrid Health Service, Madrid C/Alfonso Rodriguez Castelao 17, 28034 Madrid, Spain; rafael.aguilella@salud.madrid.org; 5 Service of Pharmacy, Center Primary Health Care Area, Madrid Health Service, Madrid C/Alberto López Palacios, 28021 Madrid, Spain; llopeze@salud.madrid.org; 6 Service of Pharmacy, Granada Metropolitano Primary Health Care Area, Andalusian Health Service, Complejo Administrativo Almanjayar C/Joaquina Eguaras nº 2, Edificio 2 Planta 1, 18013 Granada, Spain; sonia.anaya.sspa@juntadeandalucia.es (S.A.O.); marias.martin.sspa@juntadeandalucia.es (S.M.S.); 7 Primary Care Pharmacy Service, Integrated Health Organization, Barakaldo-Sestao, Osakidetza, Avenida Antonio Miranda 5, 48902 Barakaldo, Spain; ITXASNE.LECUEALCORTA@osakidetza.eus; 8 Clinical Unit Primary Care Pharmacy Area VII Asturias, Principado de Asturias Health Service, Pza. Sindicatos Mineros num 3, 33600 Mieres, Spain; jorgeignacio.jimenez@sespa.es; 9 Service of Pharmacy, Burgos Primary Health Care, Castilla y León Health, Burgos, Castilla y León, C/Calle José María de la Puente, 09006 Burgos, Spain; mafernandezvi@saludcastillayleon.es (M.F.V.); cmarquina@saludcastillayleon.es (C.M.V.); 10 Cantabria Primary Care Management, Cantabrian Health Service, C/Vargas nº 57 7 planta, 39010 Santander, Spain; yolandaborrego77@hotmail.com (Y.B.I.); raquel.prieto@scsalud.es (R.P.S.); silvia.casado@scsalud.es (S.C.C.); 11 Service of Pharmacy, Mutua Terrasa, Barcelona, CAP Sant Cugat, C/de la Mina s/n, 08173 Sant Cugat del Vallès, Spain; rmadridejos@mutuaterrassa.es (R.M.); rtomas@mutuaterrassa.es (R.T.S.); sgallardo@mutuaterrassa.es (S.G.B.); 12 Primary Care Pharmacy and Residential Social Centers, Cantabrian Health Service, Santander, Cantabria, Av Herrera Oria s/n, 39011 Santander, Spain; maria.oro@scsalud.es; 13 Clinical Unit Primary Care Pharmacy Area, Area VIII-Asturias, Principado de Asturias Health, Asturias, C/Manuel Suarez s/n, 33930 Langreo, Spain; lazarolopezeva@gmail.com; 14 Service of Primary Care Pharmacy, Zaragoza II Sector, Aragón Health Service, Zaragoza, C/Condes de Aragón 30, 50009 Zaragoza, Spain; mbpina@salud.aragon.es (M.B.P.G.); mjlallana@salud.aragon.es (Á.M.J.L.); 15 San Roque Health Center, Lugo Health Area, Cervo Monforte, Rúa Peña Anda 1, 27002 Lugo, Spain; Maria.Mercedes.Pereira.Pia@sergas.es; 16 Condado-Campiña Health District, La Palma del Condado, C/Ronda de los Legionarios 8, 21700 Huelva, Spain; mariavmaestre@gmail.com; 17 Unit of Pharmacy, Lerida Primary Care Direction, Lerida Territorial Management, Catalan Institute of Health, Lerida, Cataluña, Avenida de Blondel 23, 25002 Lleida, Spain; eribes.lleida.ics@gencat.cat; 18 Clinical Unit Primary Care Pharmacy Area V, Asturias, Principado de Asturias Health Service, Gijón, Asturias C/Severo Ochoa s/n, 33208 Gijón, Spain; constanzago2016@gmail.com; 19 Pharmacy Sub-Directorate, Navarre Health Service, Pamplona, Plaza de la Paz s/n, Planta 4, 31002 Pamplona, Spain; mc.celaya.lecea@navarra.es; 20 Service of Pharmacy, Valladolid Primary Health Care, Castilla y Leon Health, Valladolid, Castilla y León, Cardenal Torquemada nº 54, 47010 Valladolid, Spain; maprpr@gmail.com; 21 Mérida Health Area Management, Merida Hospital, Badajoz, Extremadura, Av. Don Antonio Campos Hoyos 26, 06800 Mérida, Spain; arancha.aranguez@salud-juntaex.es; 22 Service of Pharmacy, Gran Canaria Health Area, Canary Islands Health Service, C/Luis Doreste Silva 34-40, Canary Islands Health Service, 35004 Las Palmas de Gran Canaria, Spain; volmqui@gobiernodecanarias.com; 23 Primary Care Barcelona City, Catalan Institute of Health, Barcelona, C/Manso 19, 1ª Planta, 08015 Barcelona, Spain; nvillenr.bcn.ics@gencat.cat (N.V.R.); mlestonv.bcn.ics@gencat.cat (M.L.V.); atroncoso@gencat.cat (A.T.M.); 24 Health Management Area Campo de Gibraltar Pharmacy Unit, Pharmaceutical Primary Care, Carretera Getares, s/n, 11207 Algeciras, Spain; carolina.paya.sspa@juntadeandalucia.es; 25 Primary Care Management, Integrated Care Management Albacete-University Hospital Complex, Castilla-La Mancha Health Service, Albacete, Castilla La Mancha, C/Hermanos Falcó 37, 02006 Albacete, Spain; malloret@sescam.org; 26 Directorate of Health Care and Evaluation, Principado de Asturias Health Service, Oviedo, Asturias, Pza del Carbayón 1, 33001 Oviedo, Spain; alvaro.fernandezf@sespa.es; 27 Pharmacy Unit, Northwest Primary Health Care Area, Madrid Health Service, Madrid, Avda. De España 7, 28220 Majadahonda, Spain; blanca.basagoiti@salud.madrid.org (B.B.C.); amartina@salud.madrid.org (A.M.A.); adiez@salud.madrid.org (A.D.A.); 28 Pharmacy Service, Regional Hospital of Manacor, Balearic Islands Health Service, Inca, Balearic Islands, Carretera Manacor-Alcudia, s/n, 07500 Manacor, Spain; anaaurelia183@hotmail.com; 29 Cuenca Primary Care Management, Hospital Virgen de la Luz, Castilla La Mancha Health Service, C/Hermandad de Donantes de Sangre 1, 16002 Cuenca, Spain; emarcot@sescam.jccm.es; 30 Pharmacy Unit, Management of Urgencies and Health Emergencies, Murcia, C/Escultor José Sánchez Lozano Nº 7, 2ª planta, 30005 Murcia, Spain; mariaa.ariza@carm.es; 31 Pharmaceutical Health Area, Health Department Alicante, Valencia C/Pintor Baeza 12, (Edificio gris) 7ª planta, 03010 Alicante, Spain; aparicio_marcue@gva.es; 32 Principado de Asturias Health Service Pharmacy Coordination, Central Services, Principado de Asturias Health Service, Asturias, Pza Carbayón 1, 33001 Oviedo, Spain; marialuisa.nicieza@sespa.es; 33 Pharmacy Service, Tramuntana Sector, Inca Regional Hospital, Balearic Islands Health Service, Balearic Islands, Carretera Vella de Llubí, s/n, 07300 Inca, Spain; angelga@hcin.es; 34 Pharmacy Unit, Jerez, Northwest Coast and Sierra de Cádiz Primary Health Care Area, Jerez de la Frontera, Cádiz, C/José Luis Diez nº 14, 11402 Jerez de la Frontera, Spain; antonio.garcia.bonilla.sspa@juntadeandalucia.es; 35 Pharmaceutical Management Service, General Directorate of Health Assistance, Murciano Health Service, Murcia Edificio Habitamia I C/Central, 7-3ª Planta, 30100 Espinardo, Spain; rafael.herrero4@carm.es; 36 Talavera de la Reina Primary Care Management, Nuestra Señora del Prado Hospital, Castilla-La Mancha Health Service, Cuenca, Ctra. Madrid-Extremadura, Km 114, 45600 Toledo, Spain; varroyo@sescam.org (V.A.P.); mbdel@sescam.jccm.es (B.d.l.H.); mitofino@sescam.jccm.es (I.T.G.); 37 Pharmacy Service, Vinarós Department of Health, Castellón, Comunidad Valenciana, Av/Gil de Atrocillo s/n, 12500 Vinaròs, Spain; mateu_mon@gva.es; 38 Bierzo Health care Management, El Bierzo Hospital, Castilla y León Health Service, C/Médicos sin Frontera, 7, 24404 Ponferrada, Spain; pgarciav@saludcastillayleon.es; 39 Faculty of Social Sciences, Area of Design, Gaming and Multimedia, Madrid, Calle Tajo s/n, 28670 Villaviciosa de Odón, Spain; joaquin.perez@universidadeuropea.es; 40 Service of Pharmacy, Aljarafe-Sevilla Norte Primary Health Care Area, Avda de las Américas s/n, 41927 Mairena del Aljarafe, Spain

**Keywords:** SARS-CoV-2, COVID-19, macrolides, azithromycin, long-term treatment, outpatients, ambulatory care

## Abstract

The aim of this study was to know the prevalence and severity of COVID-19 in patients treated with long-term macrolides and to describe the factors associated with worse outcomes. A cross-sectional study was conducted in Primary Care setting. Patients with macrolides dispensed continuously from 1 October 2019 to 31 March 2020, were considered. Main outcome: diagnosis of coronavirus disease-19 (COVID-19). Secondary outcomes: symptoms, severity, characteristics of patients, comorbidities, concomitant treatments. A total of 3057 patients met the inclusion criteria. Median age: 73 (64–81) years; 55% were men; 62% smokers/ex-smokers; 56% obese/overweight. Overall, 95% of patients had chronic respiratory diseases and four comorbidities as a median. Prevalence of COVID-19: 4.8%. This was in accordance with official data during the first wave of the pandemic. The most common symptoms were respiratory: shortness of breath, cough, and pneumonia. Additionally, 53% percent of patients had mild/moderate symptoms, 28% required hospital admission, and 19% died with COVID-19. The percentage of patients hospitalized and deaths were 2.6 and 5.8 times higher, respectively, in the COVID-19 group (*p* < 0.001). There was no evidence of a beneficial effect of long-term courses of macrolides in preventing SARS-CoV-2 infection or the progression to worse outcomes in old patients with underlying chronic respiratory diseases and a high burden of comorbidity.

## 1. Introduction

The Severe Acute Respiratory Syndrome Coronavirus 2 (SARS-CoV-2) pandemic is having dramatic consequences around the world. Infection prevention is the most desirable solution. Since the beginning of the pandemic, research is under way in order to identify therapies and vaccines for coronavirus disease-19 (COVID-19). Vaccines are being a great success in the fight against this new disease. Their safety and efficacy have been studied in millions of people, although there are still no data on the duration of the protection or on how their efficacy may be influenced by the variants of SARS-CoV-2. However, not all patients may benefit from vaccines, so we cannot rely exclusively on vaccines to end the pandemic. Controlling the COVID-19 pandemic still needs multi-pronged strategies, in addition to effective vaccination and non-pharmacological prophylaxis [1].

The effectiveness of anti-inflammatory agents such as corticoids or tocilizumab and in the effectiveness of antiviral agents suggest that the management of hyperreactivity of the host immune response is advantageous to prevent mortality over targeting viral replication itself [2,3]. Macrolides are drugs widely used in the treatment of chronic inflammatory lung diseases to improve pulmonary function based on their antibacterial, antiviral, anti-inflammatory, and immunomodulatory properties [4,5,6,7,8]. Long-term macrolides treatments are commonly used in patients with chronic obstructive pulmonary disease (COPD), asthma, bronchiectasis or cystic fibrosis, since they are expected to reduce exacerbations and progression to severe disease [5,6,8,9,10,11,12,13,14,15,16,17,18].

Azithromycin was proposed as a potential therapy for the treatment of SARS-CoV-2 pneumonia at the beginning of the pandemic, due to the publication of a low-quality study with low sample size and many methodological limitations. This study showed that the treatment with hydroxychloroquine in combination with azithromycin had better results in reducing nasal viral load in patients with COVID-19 than hydroxychloroquine in monotherapy [19]. Later, it was ruled out as an option in the treatment of COVID-19 because it did not provide benefit to moderate to severe patients once the disease had progressed and required hospitalization. In addition, no effect was observed on mortality, progression to severe course or intensive care unit admission when administered in combination with hydroxychloroquine or with lopinavir-ritonavir, and on time to viral clearance [2,3,20,21,22]. When used in earlier stages of the disease, azithromycin was not beneficial in patients over 65 years (or over 50 years with at least one comorbidity), in reducing recovery time or decrease the risk of hospitalization [23]. Besides, azithromycin did not reduce the risk of hospitalization or death in patients with mild-moderate COVID-19 managed in the community [24].

These studies provide clear evidence that acute treatment with azithromycin is not an effective treatment for COVID-19 patients after exposure to the virus. However, there are still no results of clinical trials establishing whether long-term treatment with azithromycin or other macrolides could be helpful to prevent disease or worse outcomes. According to the literature, it takes up to 3 months of therapy for macrolides to show a significant effect as immunomodulatory agents and these benefits could disappear 3 months after treatment cessation [6]. Then, this supposed protective effect should be studied in patients who had been receiving macrolides continuously or cyclically for at least 3 months, before exposure to SARS-CoV-2. In clinical practice, patients receiving long courses of macrolides are characterized by their old age, underlying comorbidities and high morbidity and mortality rates [25]. The pandemic is having a higher impact on this population group, with a greater risk of worse outcomes.

The objectives of this work were: (a) to know the prevalence and severity of COVID-19 in adult patients treated with long-term macrolides in Primary Care; (b) to describe the factors that could be associated with worse outcomes.

## 2. Results

### 2.1. Study Population 

We identified 3057 patients on long-term treatment with macrolides that met the criteria established in this study.

The baseline characteristics of the study patients are shown in Table 1. The median age was 73 (64–81) years; 55% were men; around 62% were smokers or former smokers, 56% lived with obesity or overweight (BMI ≥ 25 kg/m^2^), and 3% resided in nursing homes. The great majority of patients (95%) had chronic respiratory diseases, mainly COPD, bronchiectasis, and asthma. Other common conditions were cardiovascular diseases, neurological/mental, and diabetes mellitus. As a median, they had 4 (3–5) underlying chronic comorbidities, and five risk factors for severe COVID-19.

Regarding treatment with macrolides, 98% of patients received azithromycin, 2% clarithromycin, and less than 1% erythromycin. Patients with azithromycin courses have been in treatment for more than 39 months, at a median weekly dose of 1500 mg. In addition, as a median, they received 11 concomitant treatments. As shown in Table 2, the most commonly prescribed drugs were bronchodilators, corticoids, proton-pump inhibitors (PPI), antihypertensives, analgesics, benzodiazepines, lipid-lowering agents, and antibiotics (other than macrolides)

A total of 23% were admitted to the hospital during the study period, and 4% were dead at the time of data collection.

### 2.2. Characteristics of Patients with COVID-19 

A total of 146 cases of COVID-19 were counted: 70 were confirmed cases, 59 were suspected infections, and 17 were probable infections (Table 3). The prevalence of COVID-19 was 4.8% although it widely changed among regions (Table S1, Supplementary data). Most of the cases were detected in March and April. The most common symptoms were respiratory: shortness of breath, cough, and pneumonia. As shown in Table 3, 53% of patients had mild/moderate symptoms, 28% required hospital admission, and 19% died with COVID-19.

Male sex, smokers or ex-smokers, and residents in nursing homes were more represented in the COVID-19 group. There were no differences in the number of comorbidities. However, some conditions including chronic respiratory failure, heart failure, neurological or mental diseases, liver failure, and lupus erythematosus were more prevalent in the COVID-19 group. Lung transplant was less represented among COVID-19 patients (Table 1).

Regarding treatment with macrolides, the great majority of patients (98%) received azithromycin at a weekly dose of 1500 mg in both groups. There were statistically significant differences in the duration of treatment: COVID-19 patients had been in treatment with macrolides for less time than non-COVID-19 patients (*p* < 0.031) (Table 2).

The number of concomitant treatments was higher in the COVID-19 group when compared to the non-COVID-19 group. Drugs more prescribed in COVID-19 patients were bronchodilators, systemic antiasthmatics (roflumilast), antibiotics other than macrolides (third generation cephalosporins and fluoroquinolones), antihistamines, antidepressants, antipsychotics, and hydroxychloroquine. The number of treatments considered to increase the risk of pneumonia was higher in the COVID-19 group (*p* = 0.002). The use of other common drugs, such as corticosteroids, PPI, antihypertensives, or lipid-lowering agents, antiplatelet drugs or anticoagulants did not differ by COVID-19 status.

The percentage of patients admitted to hospital during the study period and the percentage of deaths were 2.6 and 5.8 times higher, respectively, in the COVID-19 group than in the non-COVID-19 group (*p* < 0.001) (Table 1).

### 2.3. Factors Associated with Worse Outcomes in COVID-19 Patients

Patients with severe COVID-19 were older and had more comorbidities than patients with mild/moderate disease (Table 4). Male sex, COPD, bronchiectasis, cardiovascular diseases, diabetes mellitus, and chronic kidney disease were more frequent in patients with worse outcomes although only older age, number of comorbidities, and cardiovascular diseases reached statistical significance. There were no differences in the duration of treatment with macrolides neither in the number of concomitant treatments nor in the type of prescribed drugs between severe and non-severe COVID-19 patients (Table 5).

Regarding recorded symptoms, 27.0% of patients with cough, 38.4% with shortness of breath, and 58.5% with pneumonia were admitted to the hospital. In addition, 14.3% of patients with cough, 22.1% with shortness of breath, and 36.6% with pneumonia died.

## 3. Discussion

In this nationwide study, we studied a cohort of patients in treatment with long-term courses of macrolides during the first wave of COVID-19 in Spain. Patients included in this study were characterized by their old age, underlying chronic respiratory diseases, and multiple chronic conditions such as cardiovascular diseases or hypertension, in addition to their respiratory disease. We found a prevalence of COVID-19 infection of 4.8%. This was in accordance with data from the Spanish Ministry of Health, Consumer Affairs and Social Welfare during the first wave of pandemic (5.0–5.2%) [26], with similar variability between regions and the peak in numbers of people affected in March and April 2020 [27,28].

Patients with chronic conditions, like those described in this study, deal with colonization, frequent infections and exacerbations, a high rate of *Pseudomonas aeruginosa*, and multidrug resistant bacteria [25]. These facts make them more susceptible to a poor prognosis and a higher probability of hospitalization and death regardless of COVID-19 [25,29]. The convergence of the threat of COVID-19 and antimicrobial resistance is a cocktail for devastating effects for this population [28,30,31,32,33,34,35,36,37,38]. Being a very high-risk population, it is supposed that they have probably not left their homes, and have taken strict protective measures against infections: social distancing, the use of face mask and hydroalcoholic disinfectant gel, and strict control of symptoms with medication. This may have helped to prevent exacerbations or worsening of their baseline situation. This would justify the fact that patients in the study who did not get COVID-19, showed a lower degree of hospitalization (21% vs. 30%) and mortality (3% vs. 6%) when compared to the same population group studied 3 years before the pandemic in the same setting [25].

Regarding patients who got COVID-19, they were more frequently male, smokers or ex-smokers, and residents in nursing homes. There were no differences in age, number of comorbidities or the presence of chronic respiratory diseases between COVID-19 and non-COVID-19 patients. However, the greater therapeutic burden, the higher use of bronchodilators, other systemic antiasthmatics, and anticoagulants suggest a higher severity in their underlying pathology. It should be noted that patients who acquired COVID-19 had been in treatment with macrolides for less time than those who did not acquire the infection (*p* = 0.031). Given the methodology of the study, we cannot delve further into this observation as further studies are needed to clarify this finding in patients with a longer treatment period with macrolides before SARS-CoV-2 exposition.

The most common reported symptoms in COVID-19 patients were respiratory (shortness of breath, cough, and pneumonia), which are in turn, common in these patients due to their underlying pathology. Despite long-term treatment with macrolides, and the concomitant treatment with other groups of antibiotics in 38% of patients, pneumonia was reported in 28% of COVID-19 cases. Pneumonia was the symptom most frequently associated with hospital admissions (58.5% of patients) and death with COVID-19 (36.6% of patients).

Antibiotics have shown little therapeutic value in COVID-19, due to the low frequency of bacterial co-infections [39,40,41]. Their use in the patients in our study would be justified by an attempt to minimize the risk of exacerbations or recurrence of severe bacterial infections that could aggravate the symptoms of COVID-19. However, we did not observe differences in the use of antibiotics (other than macrolides) between patients who progressed to worse outcomes and those who did not. Treatment of high-risk patients with antibiotics in our setting did not have an impact on the prevention of severe COVID-19 outcomes, including pneumonia. Regarding the greater use of hydroxychloroquine, it could be explained by its widespread use during the first months of the pandemic, although there was no clear evidence of benefit [42].

The rate of hospital admissions in COVID-19 patients in our study was lower in comparison with data shown in official national reports (28% vs. 38–45%) [27,28]. By contrast, case fatality rate was higher(19% vs. 8–12%) [27,28]. Mortality is a more reliable outcome to assess severity, since some elderly patients who would have required admission to hospital may not have been admitted because of the high pressure of the healthcare system in Spain [28]. When compared to the patients on long-term treatment with macrolides who did not acquire the infection, COVID-19 patients were 3 times more likely to be admitted to hospital and 6 times more likely to die (*p* < 0.001).

According to the available literature [28,31,34,35,36,37,38,43], patients with severe outcomes of COVID-19 were older, male and presented more comorbidities including cardiovascular and cerebrovascular diseases, COPD, bronchiectasis, malignancies, diabetes mellitus, chronic kidney failure and liver failure, and less frequently asthma. However, statistically significant differences were only observed for age, the number of comorbidities and the presence of cardiovascular and cerebrovascular diseases. No statistically significant differences were found in other variables, probably due to the small number of patients.

Given the data, there does not seem to be a different behavior against infection in patients on long-term treatment with macrolides regarding the acquisition of infection or the development of worse outcomes. The morbidity and mortality of our study patients seem to be explained by the same factors as patients who do not receive long-term macrolides treatment.

This study has several strengths. First, it includes a large representative sample of the national territory, including all adult patients cared for by the Public Health Care Services. Full information about the patient’s characteristics, underlying pathologies, and medication burden were individually collected. The degree of under-registration of diagnoses in medical records was very low compared to other studies conducted in Primary Care, which reported 30–60% diagnoses unknown [44].

Second, it is necessary to generate more knowledge in Primary Care. The great majority of the COVID-19 research has been conducted in the hospital setting. More studies are needed in Primary Care, since even for very high-risk patients, such as those included in this study, care/coverage is mostly carried out from this setting (71% of the study patients were followed up by Primary Care). The use of Primary Care data could help to identify characteristics, evolution risk factors for severe COVID-19 of patients who were not admitted to hospitals.

Third, in contrast with other studies conducted with short-term treatments, the potential anti-inflammatory effect of macrolides (azithromycin) was assessed when administered from long-term before exposure to SARS-CoV-2.

Several limitations of the study should be pointed out. First, it is an observational study. It does not allow us to establish causal associations of the observations. Regarding the control group, there were no data from patients with the same characteristics who have not received long-term macrolides. As an external comparator, we used the Spanish official data. On the other hand, the control group for patients who acquired COVID-19 in the study was the group of patients who did not acquire the disease. Second, patients were included based on medication prescriptions. The ascertainment of medication use from the electronic health record may not reflect real exposure. We had to assume a good therapeutic adherence, based on the pharmacy records of dispensed drugs. Third, we did not have access to hospital records. Information about other symptoms, degree of severity, data on microbiology or treatments may have been under-reported in Primary Care Digital Health History. Fourth, some patients who became symptomatic since the beginning of March were diagnosed based on symptoms because this reflects Primary Care settings, where timely testing might not have been available to all patients particularly at the early stages of the pandemic (testing capacity was limited to patients who needed hospital admission or living in nursing homes). Until mid-April, SARS-CoV-2 PCR or serology diagnostics were not available in Primary Care Health Centers. Even so, 48% of patients were confirmed cases with molecular diagnostic techniques, a higher value compared to other studies conducted in Primary Care [31]. Fifth, there may be additional confounders influencing outcomes. For example, patients in this study have a high burden of diseases and comorbidities. Multimorbidity and polypharmacy make it challenging to interpret the results, especially in this case, in which patients present chronic respiratory diseases, whose symptoms may be confused with those of COVID-19.

## 4. Materials and Methods

### 4.1. Design

A descriptive cross-sectional multicenter study was carried out in the Primary Care setting. The study comprised 47 Healthcare Areas from 16 of the 17 regions in Spain, covering a population of 14,349,076 people (Table S1, Supplementary data).

Patients ≥18 years of age assigned to Primary Care Centers from the study areas, who had been prescribed and dispensed in community pharmacy at least 10 packages of azithromycin (J01FA10), five packages of clarithromycin (J01FA09) or five packages of erythromycin (J01FA01) for systemic use (according to the WHO Anatomical Therapeutic Chemical classification system) [45], from 1 October 2019 to 31 March 2020 were considered. These agents were selected as the immunomodulatory actions were attributed to 14- and 15-membered macrolides [6].

The exclusion criteria were: (a) treatment with macrolides started after 31 October 2019; (b) treatment was completed before 29 February 2020; (c) there was any indication that the patient had not received macrolides in a continuous manner, as prescribed; (d) patients without data available in the electronic medical records or those with private pharmacy. The aim was to select patients who were on treatment with macrolides for at least 6 months at the beginning of the pandemic, since it takes up to 3 months of therapy for macrolides to show a supposed significant effect as immunomodulatory agents [6].

### 4.2. Data Sources and Outcomes 

Patients were identified through databases from computerized pharmacy records of reimbursed and dispensed drugs, from their Regional Health Care Services. Individual clinical data, diagnoses, and microbiological tests from patients were collected from the electronic medical records of Public Health Services maintained for routine healthcare activities. Population data were obtained from the Statistics National Institute [46].

Data were collected between 15 July 2020 and 30 September 2020. An electronic form, with restricted online access to researchers was designed ad hoc for the data collection. The anonymity of participants was guaranteed by their identification in the electronic form through a numerical code. Data were stored securely in a data center with perimeter security.

The primary outcome was defined as a positive diagnosis of COVID-19.Other outcomes related to COVID-19 disease, were: date of COVID-19 infection diagnosis, symptoms developed: unspecific (fever, headache, muscle pain), respiratory (cough, sore throat, pneumonia, shortness of breath, anosmia, ageusia, odynophagia), dermatological, gastrointestinal, and acute kidney failure. The factors that determined severity were hospitalization and death within the study period.

The data of the final follow-up was 31 May 2020. Patients were classified according to the record that appeared in the Clinical History at the time of data collection. As polymerase chain reaction technique (PCR) and serology diagnostics were not available in the early stages of the pandemic, many patients were diagnosed based on symptoms. The criteria applied for the definition of cases (confirmed: active or past infection; suspected infection; probable infection) were those defined in the latest available protocol of the Spanish Ministry of Health, Consumer Affairs and Social Welfare at the time of the study [47].Due to the continuous adaptation of the protocols regarding the registration of episodes related to COVID-19, the definition of cases in the current protocol at the time of the study is described in Table 6 [47].

The following variables related to demographic and clinical information were collected: age, gender, smoking behavior (current smoking or former smoker), obesity or high body-mass index (BMI) (BMI > 25 kg/m^2^), residence in nursing home or in long-term care facilities, the number and type of comorbidities (Table S2, Supplementary data), number of severe COVID-19 risk factors (defined as the sum of: age ≥60 years, arterial hypertension, cardiovascular disease, diabetes, chronic renal failure, obesity, respiratory chronic disease, malignancy, chronic neurological or mental diseases, liver disease, immunosuppression) [48], hospital admissions, death, concomitant therapies (Table S3, Supplementary data), and number of treatments that increase the risk of pneumonia (antipsychotics, antihistamines, antidepressants, opioids, benzodiazepines, proton-pump inhibitors, immunosuppressive agents or gabapentinoids) [49].Regarding treatment with macrolides, the following variables were recorded: macrolide agent, days on treatment with macrolides, and average weekly dose.

### 4.3. Statistical Analysis 

A descriptive analysis of the data was carried out to determine the prevalence and profile of patients. Summary statistics were computed using frequencies and percentages for categorical variables and median (50th percentile), and interquartile range (25–75th percentiles) for continuous variables showing asymmetric distribution. Qualitative variables were expressed as percentages and quantitative variables as mean and standard deviation (SD). Confidence intervals were calculated at 95% (95% CI).

A bivariate analysis was performed, followed by the calculation of a hypothesis contrast tests appropriate to the nature of the variables. Comparisons of characteristics among patients were analyzed by the Chi-square test for qualitative variables, except when some of the expected values were under 5, where the Fisher exact test was applied. For quantitative variables, we used Student’s test or U-Mann–Whitney based upon their application criteria.

The level of statistical significance has been set at a *p*-value less than 0.05. STATA Corp. V14 was used for statistical analysis (Stata Corp, College Station, 2015).

## 5. Conclusions

Our findings suggest that there is no beneficial effect of long-term courses of macrolides in preventing SARS-CoV-2 infection or the progression to worse outcomes of COVID-19 in old patients with underlying chronic respiratory diseases and a high burden of comorbidity. Further studies should be performed to confirm these results. Meanwhile, our data can be added to the set of studies showing that the antibacterial effects of azithromycin are unlikely to translate into a significant clinical benefit in COVID-19.

## Figures and Tables

**Table 1:** Socio-demographic and baseline clinical characteristics of patients.

Patient’s Characteristics	Total Patients (n, %, Median, Interquartile Range)	Non-COVID-19 Patients (n, %, Median, Interquartile Range)	COVID-19 Patients (n, %, Median, Interquartile Range)	*p*-Value
Total	3057 (100)	2911 (100)	146 (100)	
Age (years, median)	73 (64–81)	73 (64–81)	74 (64–74)	0.164
Sex (n, %men)	1687 (55.2)	1596 (54.8)	91 (62.3)	0.049
Smokers or former smokers ^a^	1611 (62.1)	1516 (61.5)	95 (72.5)	0.010
Obesity of high body-mass index (BMI ≥ 25 kg/m^2^) ^b^	1361(55.5)	1297 (55.9)	64 (49.2)	0.158
Residence in nursing homes or long-term care facilities ^c^	98 (3.3)	74 (2.6)	24 (16.4)	0.000
Number of comorbidities, median	4 (3–5)	4 (3–5)	4 (3–5)	0.904
Respiratory chronic diseases:	2917 (95.4)	2775 (95.3)	142 (97.3)	0.276
Chronic Obstructive Pulmonary Disease	1683 (55.1)	1596 (54.8)	87 (59.6)	0.259
Bronchiectasis	1091 (35.7)	1042 (35.8)	49 (36.6)	0.583
Asthma	709 (23.2)	673 (23.1)	36 (24.7)	0.667
Chronic respiratory failure	308 (10.1)	286 (9.8)	22 (15.1)	0.040
Chronic bronchitis	253 (8.3)	241 (8.3)	12 (8.3)	0.980
Emphysema	227 (7.4)	219 (7.5)	8 (5.5)	0.358
Lung transplant	200 (6.5)	197 (6.8)	3 (2.1)	0.025
Cystic fibrosis	123 (4.0)	118 (4.1)	5 (3.4)	0.706
Other	527 (17.2)	502 (17.2)	25 (17.2)	0.970
Arterial hypertension Cardiovascular, cerebrovascular diseases: Cardiovascular disease Heart failure Acute myocardial infarction Stable coronary heart disease Angina pectoris Stroke Peripheral arterial disease Transient ischemic attack Other	2001 (65.5) 1231 (40.3) 618 (20.2) 408 (13.4) 150 (4.9) 87 (2.9) 59 (1.9) 125 (4.1) 108 (3.5) 81 (2.7) 659 (21.6)	1896 (65.1) 1158 (39.8) 576 (19.8) 375 (13.9) 141 (4.8) 82 (2.8) 56 (1.9) 116 (4.0) 100 (3.4) 76 (2.6) 623 (21.4)	105 (75.9) 73 (50.0) 42(28.8) 33 (22.6) 9 (6.2) 5 (3.4) 3 (2.1) 9 (6.2) 8 (5.5) 5 (3.4) 36 (24.7)	0.092 0.014 0.008 0.001 0.471 0.192 0.911 0.194 0.192 0.550 0.351
Chronic neurological or mental diseases:	964 (31.5)	905 (31.1)	59 (40.4)	0.018
Depression	566 (18.5)	533 (18.3)	33 (22.6)	0.193
Dementia	104 (3.4)	100 (3.4)	4 (2.7)	0.651
Parkinson	42 (1.4)	40 (1.4)	2 (1.4)	0.997
Alzheimer	31 (1.0)	28 (1.0)	3 (2.1)	0.198
Schizophrenia	16 (0.5)	15 (0.5)	1 (0.7)	0.782
Other	355 (11.6)	332 (11.4)	23 (15.8)	0.110
Situation that leads to immunosuppression:	753 (24.6)	722 (24.8)	31 (21.2)	0.329
Malignancy	528 (17.3)	504 (17.3)	24 (16.4)	0.785
Transplant	174 (5.7)	168 (5.8)	6 (4.1)	0.398
Prolonged use of corticoids	45 (1.5)	42 (1.4)	3 (2.1)	0.549
Human immunodeficiency virus infection	20 (0.7)	20 (0.7)	0 (0.0)	0.315
Other	52 (1.7)	48(1.7)	4(2.7)	0.320
Autoimmune disease:	295 (9.7)	284 (9.8)	11 (7.5)	0.375
Rheumatoid arthritis	98 (3.2)	96 (3.3)	2 (1.4)	0.197
Psoriasis	61 (2.0)	56 (1.9)	5 (3.4)	0.206
Inflammatory bowel disease	34 (1.1)	32 (1.1)	2 (1.2)	0.761
Sjögren Syndrome	23 (0.8)	23 (0.8)	0 (0.0)	0.281
Lupus erythematosus	19 (0.6)	16 (0.6)	3 (2.1)	0.024
Celiac disease	4 (0.1)	4 (0.1)	0 (0.0)	0.110
Multiple sclerosis	3 (0.1)	3 (0.1)	0 (0.0)	0.698
Other	82 (2.7)	82 (2.8)	0 (0.0)	0.040
Other conditions				
Diabetes mellitus	766 (25.1)	731(25.1)	35(24.0)	0.757
Chronic kidney failure	372 (12.2)	352 (12.1)	20 (13.7)	0.562
Liver disease or failure	137 (4.5)	122 (4.2)	15 (10.3)	0.001
Hospital admissions (for any cause)	684 (22.6)	605 (21.0)	79 (54.5)	0.000
Death	120 (3.9)	93 (3.2)	27 (18.5)	0.000

^a^ Data from 2596 patients. ^b^ Data from 2452 patients. ^c^ Data from 3014 patients.

**Table 2:** Treatments with macrolides and concomitant treatments.

Patient’s Treatments	Total Patients (n, %, Median, Interquartile Range)	Non-COVID-19 Patients (n, %, Median, Interquartile Range)	COVID-19 Patients (n, %, Median, Interquartile Range)	*p*-Value
Total	3057 (100)	2911 (100)	146 (100)	
**Treatment with long-term macrolides**				
Number of patients				
Azithromycin	2987 (97.7)	2842 (97.6)	145 (99.3)	0.184
Clarithromycin	55 (1.8)	53 (1.8)	2 (1.4)	0.689
Erythromycin	16 (0.5)	16 (0.6)	-	-
Days with macrolides ^a^, median				
Azithromycin	580 (324–1123)	584 (327–1124)	468 (269–1070)	0.031
Clarithromycin	354 (234–578)	354 (231–542)	-	-
Erythromycin	456 (260–702)	456 (260–702)	-	-
Weekly dose (mg), median				
Azithromycin	1500 (750–1500)	1500 (750–1500)	1500 (1000–1500)	0.487
Clarithromycin	7000 (3500–7000)	7000 (3500–7000)	-	-
Erythromycin	2300 (1050–3500)	2300 (1050–3500)	-	-
**Current medication**				
Number of concomitant treatments	11 (±4)	11 (±4)	12 (±5)	0.000
Bronchodilators:	2600 (85.1)	2467 (84.8)	133 (91.1)	0.036
Long-acting β2-agonist (LABA)	2279 (74.6)	2162 (74.3)	117 (80.1)	0.112
Long-acting muscarinic antagonist (LAMA)	1824 (59.7)	1728 (59.4)	96 (65.8)	0.124
Short-acting β2-agonist (SABA)	1431 (46.8)	1361 (46.8)	70 (47.8)	0.778
Short-acting muscarinic antagonist (SAMA)	745 (24.4)	705 (24.2)	40 (27.4)	0.383
Othersystemicantiasthmatics:	533 (17.4)	496 (17.1)	37 (25.3)	0.010
Montelukast	390 (12.8)	367 (12.6)	23 (15.8)	0.266
Roflumilast	127 (4.2)	113 (3.9)	14 (4.2)	0.001
Omalizumab	10 (0.33)	9 (0.31)	1 (0.68)	0.438
Other	29 (1.0)	27 (0.9)	2 (1.4)	0.591
Corticoids:	2491 (81.5)	2369 (81.4)	122 (83.6)	0.508
Inhaled	2158 (70.6)	2045 (70.3)	113 (77.4)	0.064
Systemic	870 (28.5)	829 (28.5)	41 (28.1)	0.918
Protonpumpinhibitors	2247 (73.5)	2134 (73.3)	113 (77.4)	0.275
Antihypertensives	1919 (62.8)	1822 (62.6)	97 (66.4)	0.348
Analgesics:	1842 (60.3)	1750 (60.1)	92 (63.0)	0.485
Non-opioids	1644 (53.8)	1560 (53.6)	84 (57.5)	0.351
Opioids	660 (21.6)	631 (21.7)	29 (19.9)	0.603
Gabapentinoids	290 (9.5)	275 (9.5)	15 (10.3)	0.739
Benzodiazepines	1265 (41.4)	1198 (41.2)	67 (45.9)	0.257
Lipid-loweringagents	1225 (40.1)	1162 (39.9)	63 (43.2)	0.437
Antibiotics (otherthanmacrolides)	893 (29.2)	837 (28.8)	56 (38.4)	0.013
Fluoroquinolones	387 (12.7)	350 (12.0)	37 (25.3)	0.000
Levofloxacin	242 (7.9)	210 (7.2)	32 (21.9)	0.000
Ciprofloxacin	133 (4.4)	125 (4.3)	8 (5.5)	0.493
Moxifloxacin	58 (1.9)	54 (1.9)	4 (2.7)	0.445
Penicillins	197 (6.4)	188 (6.5)	9 (6.2)	0.888
Amoxicillin-clavulanate	167 (5.5)	160 (5.5)	7 (4.8)	0.716
Amoxicillin	35 (1.1)	33 (1.1)	2 (1.2)	0.793
Cephalosporins	94 (3.1)	79 (2.7)	15 (10.3)	0.000
Cefditoren	47 (1.5)	41 (1.4)	6 (4.1)	0.010
Cefuroxime	37 (1.2)	35 (1.2)	2 (1.4)	0.857
Cefixime	7 (0.2)	5 (0.2)	2 (1.4)	0.003
Ceftriaxone	7 (0.2)	1 (0.03)	6 (4.1)	0.000
Otherantibiotics	473 (15.5)	452 (15.5)	21 (14.4)	0.709
Co-trimoxazole	256 (8.4)	247 (8.5)	9 (6.2)	0.323
Lincosamides-clindamycin	11 (0.4)	10 (0.3)	1 (0.7)	0.501
Other	255 (8.3)	242 (8.3)	13 (8.9)	0.801
Antidepressants	855 (28.0)	795 (27.3)	60 (41.1)	0.000
Antidiabetics	656 (21.5)	625 (21.5)	31 (21.2)	0.946
Antiplateletdrugs	638 (20.9)	606 (20.8)	32 (21.9)	0.750
Anticoagulants	554 (18.1)	519 (17.8)	35 (24.0)	0.060
NSAIDs	512 (16.8)	489 (16.8)	23 (15.8)	0.741
Antihistamines	362 (11.8)	337 (11.6)	25 (17.1)	0.043
Immunosuppressants	321 (10.5)	312 (10.7)	9 (6.2)	0.080
Mucolytics	306 (10.0)	289 (9.9)	17 (11.6)	0.500
Antipsychotics	182 (6.0)	167 (5.7)	15 (10.4)	0.024
Antifungals	165 (5.4)	155 (5.3)	10 (6.9)	0.426
Hydroxychloroquine	37 (1.2)	29 (1.0)	8 (5.5)	0.000
Coughsuppressants	27 (0.9)	24 (0.8)	3 (2.1)	0.136
Number of treatments that increase the risk of pneumonia ^b^	2 (1–4)	2 (1–4)	3 (2–4)	0.020

^a^ Until 31 May2020.^. b^ Treatments that increase the risk of pneumonia: antipsychotics, antihistamines, antidepressants, opioids, benzodiazepines, proton-pump inhibitors, immune suppressive agents, gabapentinoids.

**Table 3:** Clinical characteristics of COVID-19 patients.

Patient’s Characteristics	Number of Patients (n = 146, %)	95% Confidence Interval
Diagnosis of COVID-19: Confirmed Suspected Probable	70 (47.9) 59 (40.4) 17 (11.6)	(39.6–56.4) (32.4–48.8) (6.9–18.9)
Date of COVID-19 diagnosis record: February March April May	2 (1.4) 53 (36.3) 58 (39.7) 33 (22.6)	(0.2–4.9) (28.5–44.7) (31.7–48.1) (16.1–30.3)
COVID-19 symptoms: Asymptomatic Unspecific * Respiratory: Shortness of breath Cough Pneumonia Anosmia, ageusia Other Gastrointestinal Dermatological Acute kidney failure Other	13 (8.9) 5 (3.4) 121 (82.9) 86 (58.9) 63 (43.2) 41 (28.1) 5 (3.4) 8 (5.5) 17 (11.6) 3 (2.1) 2 (1.4) 7 (4.8)	(4.8–14.7) (1.1–7.8) (75.8–88.6) (50.5–67.0) (35.0–51.6) (21.0–36.1) (1.1–7.8) (2.4–10.1) (6.9–18.9) (0.4–5.9) (0.2–4.9) (1.9–9.6)
Severity: Mild to moderate Hospitalization with COVID-19 Death	78 (53.4) 41 (28.1) 27 (18.5)	(45.0–61.7) (21.0–36.1) (12.6–25.8)

* Unspecific: fever, headache, muscle pains, fatigue.

**Table 4:** Socio-demographic and baseline clinical characteristics of patients with COVID-19 by severity.

Patient’s characteristics	Total Patients (*n*, %, Median, Interquartile Range)	Mild/Moderate (*n*, %, Median, Interquartile Range)	Hospitalization/Death (*n*, %, Median, Interquartile Range)	*p*-Value
Total	146 (100)	78 (53.4)	68 (46.6)	
Age (years, median)	74 (64–74)	71 (63–78)	81 (69–86)	0.004
Sex (n, %men)	91 (62.3)	44 (56.4)	47 (69.1)	0.114
Smokers or ex-smokers ^a^	95 (72,5)	50 (73.5)	45 (71.4)	0.788
Obesity of high body-mass index (BMI≥25 kg/m^2^) ^b^	64 (49.2)	35 (51.5)	29 (46.8)	0.593
Residence in nursing homes or long-term care facilities	24 (16.4)	9 (11.5)	15 (22.1)	0.087
Number of comorbidities, median	4 (3–5)	4 (2–5)	5 (4–6)	0.014
Respiratory chronic diseases:	142 (97.3)	75 (96.1)	67 (98.5)	0.380
Chronic Obstructive Pulmonary Disease	87 (59.6)	42 (53.8)	45 (66.2)	0.130
Bronchiectasis	49 (36.6)	23 (29.5)	26 (38.2)	0.264
Asthma	36 (24.7)	21 (26.9)	15 (22.1)	0.496
Chronic respiratory failure	22 (15.1)	11 (14.1)	11 (16.1)	0.727
Chronic bronchitis	12 (8.3)	8 (10.3)	4 (5.9)	0.337
Emphysema	8 (5.5)	4 (5.1)	4 (5.9)	0.842
Other	30 (20.6)	19 (24.4)	11 (16.2)	0.222
Arterial hypertension Cardiovascular, cerebrovascular diseases: Cardiovascular disease Heart failure Acute myocardial infarction Stable coronary heart disease Angina pectoris Stroke Peripheral arterial disease Transient ischemic attack Other	105 (75.9) 73 (50.0) 42(28.8) 33 (22.6) 9 (6.2) 5 (3.4) 3 (2.1) 9 (6.2) 8 (5.5) 5 (3.4) 36 (24.7)	56 (71.8) 33 (42.3) 22 (32.4) 15 (19.2) 4 (5.1) 4 (5.1) 1 (1.3) 6 (7.7) 5 (6.4) 1 (1.3) 14 (18.0)	49 (72.1) 40 (58.8) 20 (25.6) 18 (26.5) 5 (7.4) 1 (1.5) 2 (2.9) 3 (4.4) 3 (4.4) 4 (5.9) 22 (32.3)	0.972 0.046 0.371 0.297 0.577 0.225 0.481 0.127 0.597 0.411 0.044
Chronic neurological or mental diseases	59 (40.4)	31 (39.7)	28 (41.2)	0.860
Situation that leads to immunosuppression:	31 (21.2)	13 (16.7)	18 (26.5)	0.148
Malignancy	24 (16.4)	11 (14.1)	13 (19.1)	0.415
Transplant	6 (4.1)	2 (2.6)	4 (5.9)	0.314
Other	6 (4.1)	1 (1.3)	5 (7.4)	0.065
Autoimmune disease	11 (7.5)	9 (11.5)	2 (2.9)	0.050
Other conditions				
Diabetes mellitus	35 (24.0)	15 (19.2)	20 (29.4)	0.151
Chronic kidney failure	20 (13.7)	7 (9.0)	13 (19.1)	0.075
Liver disease or failure	15 (10.3)	6 (7.7)	9 (13.2)	0.271
Number of severe COVID-19 risk factors ^c^, mean (±SD)	6 (±2)	5 (±2)	6 (±2)	0.082

^a^ Data from 131 patients. ^b^ Data from 130 patients. ^c^ Risk factors for severe COVID-19:≥60 years, arterial hypertension, cardiovascular disease, diabetes, chronic renal failure, obesity, respiratory chronic disease, malignancy, chronic neurological or mental diseases, liver disease, immune suppression.

**Table 5:** Treatments with macrolides and concomitant treatmentsin patients with COVID-19 by severity.

Patient’s Treatment	Total Patients (*n*, %, Median, Interquartile Range)	Mild/Moderate (*n*, %, Median, Interquartile Range)	Hospitalization/Death (*n*, %, Median, Interquartile Range)	*p*-Value
Total	146 (100)	78 (53.4)	68 (46.6)	
Treatment with long-term macrolides (azythromycin)				
Number of patients	145 (99.3)	77 (98.7)	68 (100)	0.349
Days with macrolides ^a^, median	468 (269–1070)	468 (273–936)	499 (257–1127)	0.934
Weekly dose (mg), median	1500 (1000–1500)	1500 (1000–1500)	1500 (1000–1500)	0.584
**Current medication**				
Number of concomitant treatments	12 (±5)	11 (±5)	13 (±4)	0.059
Bronchodilators	133 (91.1)	69 (88.5)	64 (94.1)	0.231
Long-acting β2-agonist (LABA)	117 (80.1)	62 (79.5)	55 (80.9)	0.833
Long-acting muscarinic antagonist (LAMA)	96 (65.8)	51 (65.4)	45 (66.2)	0.920
Short-acting β2-agonist (SABA)	70 (47.8)	34 (43.6)	36 (52.9)	0.259
Short-acting muscarinic antagonist (SAMA)	40 (27.4)	18 (23.1)	22 (32.4)	0.210
Other systemic antiasthmatics	37 (25.3)	20 (25.6)	17 (25.0)	0.929
Montelukast	23 (15.8)	13 (16.7)	10 (14.7)	0.746
Roflumilast	14 (4.2)	7 (9.0)	7 (10.3)	0.787
Corticoids:	122 (83.6)	63 (80.8)	59 (86.8)	0.330
Inhaled	113 (77.4)	59 (75.6)	54 (79.4)	0.587
Systemic	41 (28.1)	18 (23.1)	23 (33.8)	0.149
Proton pump inhibitors	113 (77.4)	57 (73.1)	56 (82.4)	0.181
Antihypertensives	97 (66.4)	51 (65.4)	46 (67.7)	0.773
Analgesics:	92 (63.0)	45 (57.7)	47 (69.1)	0.154
Non-opioids	84 (57.5)	41 (52.6)	43 (63.2)	0.193
Opioids	29 (19.9)	17 (21.8)	12 (17.7)	0.531
Gabapentinoids	15 (10.3)	9 (11.5)	6 (8.8)	0.590
Benzodiazepines	67 (45.9)	39 (50.0)	28 (41.2)	0.286
Lipid-loweringagents	63 (43.2)	33 (42.3)	30 (44.1)	0.826
Antibiotics (other than macrolides)	56 (38.4)	30 (38.5)	26 (38.2)	0.978
Fluoroquinolones	37 (25.3)	20 (25.6)	17 (25.0)	0.929
Cephalosporins	15 (10.3)	6 (7.7)	9 (13.2)	0.271
Penicillins	9 (6.2)	5 (6.4)	4 (5.9)	0.895
Other antibiotics	21 (14.4)	11 (14.1)	10 (14.7)	0.917
Antidepressants	60 (41.1)	32 (41.0)	28 (41.2)	0.985
Antidiabetics	31 (21.2)	12 (15.4)	19 (27.9)	0.064
Antiplatelet drugs	32 (21.9)	17 (21.8)	15 (22.1)	0.969
Anticoagulants	35 (24.0)	15 (19.2)	20 (29.4)	0.151
NSAIDs	23 (15.8)	14 (18.0)	9 (13.2)	0.435
Antihistamines	25 (17.1)	13 (16.7)	12 (17.7)	0.875
Immunosuppressants	9 (6.2)	2 (2.6)	7 (10.3)	0.053
Mucolytics	17 (11.6)	6 (7.7)	11 (16.2)	0.111
Antipsychotics	15 (10.4)	7 (8.8)	8 (11.8)	0.580
Antifungals	10 (6.9)	5 (6.4)	5 (7.4)	0.822
Hydroxychloroquine	8 (5.5)	3 (3.9)	5 (7.4)	0.353
Number of treatments that increase the risk of pneumonia ^b^	3 (2–4)	3 (2–4)	3 (1–4)	0.506

^a^ Until 31 May2020. ^b^ Treatments that increase the risk of pneumonia: antipsychotics, antihistamines, antidepressants, opioids, benzodiazepines, proton-pump inhibitors, immune suppressive agents, gabapentinoids.

**Table 6:** COVID-19 case definition, according to the current protocol at the time of the study [47].

Confirmed case	Any person with laboratory confirmation of SARS-CoV-2 infection by reverse-transcription PCR (PCR) test (or other molecular diagnostic technique considered appropriate), or patients that meet clinical criteria, with negative PCR or other molecular diagnostic technique considered adequate, and positive result for IgM by serology (not by rapid test).
Case under investigation (suspected case)	Person meeting clinical criteria until the PCR result is obtained. A suspected case of SARS-CoV-2 infection is any person with these clinical criteria: sudden-onset acute respiratory infection of any severity that includes symptoms compatible with COVID-19, among others: fever, cough or sensation of shortness of breath. Other symptoms such as odynophagia, anosmia, ageusia, muscle pain, diarrhea, chest pain or headaches, can also be considered symptoms of suspected SARS-CoV-2 infection.
Probable case	Person with severe acute respiratory infection with clinical and radiological criteria compatible with COVID-19, with negative PCR results or suspicious cases with inconclusive PCR.

## Data Availability

The data that support the findings of this study are available on request from the corresponding author.

## Supplementary Materials

The following are available online at https://www.mdpi.com/article/10.3390/antibiotics10091039/s1, Table S1: Participating Spanish regions and number of patients provided to study; Table S2: Comorbid conditions analyzed; Table S3: Concomitant treatments analyzed.
