# Supplementary material for: Sars-Cov-2 Infection in Patients on Long-Term Treatment with Macrolides in Spain: A National Cross-Sectional Study
Source: Antibiotics (Basel). 2021 Aug 25;10(9):1039. doi: 10.3390/antibiotics10091039 (PMC8468207; doi:10.3390/antibiotics10091039)
Supplement: Supplementary file 1 [file antibiotics-10-01039-s001.zip › antibiotics-1331806-SI.pdf]

antibiotics

MDPI

Article

Sars-Cov-2 Infection in Patients on Long-Term Treatment with Macrolides in Spain: A National Cross-Sectional Study

Carmen Marina Meseguer Barros [1], Natalia Alzueta Isturiz [2], Rita Sainz de Rozas Aparicio [3], Rafael Aguilella Vizcaíno [4], Laura López Esteban [5,†], Sonia Anaya Ordóñez [6], Itxasne Lekue Alkorta [7,‡], Salvadora Martín Suances [6], Jorge Ignacio Jiménez Arce [8], Maite Fernández Vicente [9], Yolanda Borrego Izquierdo [10], Raquel Prieto Sánchez [10], Silvia Casado Casuso [10], Rosa Madridejos [11], Carmen Marquina Verde [9,§], Rosa Tomás Sanz [11], María Oro Fernández [12], Sara Gallardo Borge [11], Eva Lázaro López [13], María Belén Pina Gadea [14], Mercedes Pereira Pía [15], María Victoria Maestre Sánchez [16], Esther Ribes Murillo [17], Constanza Gómez de Oña [18], María Jesús Lallana Álvarez [14], Concepción Celaya Lecea [19], María Ana Prado Prieto [20], Aranzazu Aranguez Ruiz [21], Vicente Olmo Quintana [22], Noemí Villén Romero [23], Carolina Payá Giner [24], Angeles Lloret Callejo [25], Alvaro Fernández Ferreiro [26], Blanca Basagoiti Carreño [27], Ana-Aurelia Iglesias Iglesias [28], Antonio Martín Alonso [27,‖], Ana Díez Alcántara [27], Esther Marco Tejón [29], Marta Lestón Vázquez [23], Mª Ángeles Ariza Copado [30], Marta Aparicio Cueva [31], Belén Escudero Vilaplana [1], Marisa Nicieza [32], Gracia Picazo Sanchiz [1], Genma María Silva Riádigos [1], Lucía Jamart Sánchez [1], Ángel García Álvarez [33], Antonio García Bonilla [34], Rafael Herrero Delicado [35], Virginia Arroyo Pineda [36], Belén de la Hija [36], Amelia Troncoso Mariño [23], Isabel Tofiño González [36], Mónica Susana Mateu García [37], Pablo García Vázquez [38], Joaquín Pérez Martín [39] and Rocío Fernández-Urrusuno [40,*,¶] on behalf of the Infectious Diseases SEFAP Team

Supplementary data

Table S1. Participating Spanish regions and number of patients provided to study.

Region	Patients		COVID-19 patients	
	N	%	N	%
Madrid	502	16.42	47	32.19
Catalonia	397	12.99	18	12.33
Andalucia	363	11.87	9	6.16
Basque Country	337	11.02	10	6.85
Cantabria	322	10.53	24	16.44
Asturias	282	9.22	3	2.05
Castile and Leon	228	7.46	9	6.16
Aragon	118	3.86	5	3.42
Castile La Mancha	110	3.6	8	5.48
Navarre	98	3.21	2	1.37
Galicia	66	2.16	1	0.68
Balearic Islands	58	1.90	2	1.37
Extremadura	51	1.67	1	0.68
Murcia	48	1.57	0	0.00
Canary Islands	44	1.44	5	3.42
Valencian Community	33	1.08	2	1.37
Total	3,057	100	146	100

antibiotics

MDPI

Table S2. Comorbid conditions analyzed.

Respiratory chronic disease:
Chronic Obstructive Pulmonary Disease (COPD).
Cystic fibrosis.
Bronchiectasis.
Chronic bronchitis.
Emphysema.
Chronic respiratory failure.
Asthma.
Lung transplant.
Arterial hypertension.
Cardiac disease. ischaemic or hypertensive heart disease. cerebrovascular disease:
Heart failure.
Acute myocardial infarction.
Angina pectoris.
Stable coronary heart disease.
Peripheral arterial disease.
Transient ischemic attack
Stroke
Chronic neurological or mental diseases:
Dementia.
Alzheimer.
Parkinson.
Schizophrenia
Depression.
Situation that leads to immunosuppression (for an underlying illness or from therapy):
Malignancy.
Transplant.
Human immunodeficiency virus infection (VIH).
Prolonged use of corticoids
Autoimmune diseases:
Rheumatoid arthritis.
Lupus erythematosus.
Psoriasis.
Celiac disease.
Inflammatory bowel disease.
Multiple sclerosis.
Sjögren.
Other conditions
Diabetes mellitus
Chronic kidney disease.
Liver disease or failure.

Table S3. Concomitant treatments analyzed.

Proton-pump inhibitors (PPI).
Non steroidal anti-inflammatory drugs (NSAIDs).
Analgesics. Non opiods, opiods
Gabapentinoids
Antihistamines.
Cough suppressants.
Mucolytics.
Inhaled corticoids.
Systemic corticoids.
Bronchodilators.
Other systemic antiasthmatics
Antidiabetics.
Antihypertensives.
Lipid-lowering agents.
Antiplatelet drugs.
Anticoagulants.
Antidepressants.
Benzodiazepines and Z drugs.
Antipsychotics.
Other immunosuppressive agents.
Systemic antibiotics:
Antifungal drugs
Antirheumatics: hidroxicloroquine
